# Severe generalised hypersensitivity reaction to topical neomycin after cataract surgery: a case report

**DOI:** 10.1186/1752-1947-2-57

**Published:** 2008-02-23

**Authors:** Imran A Ansari, Ernest Onyema

**Affiliations:** 1 Department of Ophthalmology, Princess Alexandra Hospital, Harlow, Essex, UK

## Abstract

**Introduction:**

Systemic hypersensitivity reactions to topical ophthalmic treatment occur rarely, but when they do they can be severe as highlighted by this case.

**Case presentation:**

A post-operative cataract surgery patient developed a severe and generalised hypersensitivity reaction following topical treatment with Maxitrol (Dexamethasone and Neomycin) eye drops. The patient reported a previous allergic reaction to Neomycin.

**Conclusion:**

This case report emphasises the importance of a thorough drug and allergy history when patients are seen at pre-assessment or clerked in for surgery.

## Introduction

Adverse external ocular effects of topical ophthalmic therapy have been estimated to occur in 10 % of all adverse reactions [1].

Drug-related ocular allergies are often the result of type IV hypersensitivity reactions, although type 1 and type 3 hypersensitivity reactions may also be involved [2].

Hypersensitivity to localised ocular therapy may involve a localised contact reaction which may include itching, redness, tearing, mucopurulent discharge, and papillary conjunctivitis as well as corneal involvement [3]. Dermatitis, oedema and chemosis of the eyelids and skin can also occur [4].

Anaphylactoid reactions are rare and can be the result of type 1 (immediate) hypersensitivity reactions [1]. They are not usually associated with systemic anaphylaxis but involve an acute shock syndrome that may be immunologically mediated [5].

Anaphylactoid reactions are characterised by acute itching, conjunctival hyperemia, chemosis and oedema of skin in the form of urticaria and angioedema Neomycin is an antibiotic that is often used in the form of Maxitrol (Neomycin, Polymyxin B and Dexamethasone) after cataract surgery in the prophylaxis of infection.

When used in ophthalmic preparations, it is well known to be a cause of allergic localised contact reactions, but it is rare for it to cause a diffuse cutaneous systemic reaction [6]. One study has estimated the incidence of allergic contact reactions to topical neomycin as 1 to 29/100,000 [7]. We describe a case of severe systemic allergy with a diffuse cutaneous hypersensitivity reaction following topical ophthalmic administration of neomycin in the form of Maxitrol.

## Case presentation

An 80 year old Caucasian male underwent routine phacoemulsification cataract extraction with posterior chamber intraocular lens implant.

The patient had a history of hypertension and hypercholesterolemia for which he was using Atenolol and Simvastatin. He also had a history of bladder cancer for which he had received chemotherapy and radiotherapy.

Of note, the patient had reported an allergic reaction to Neomycin ear drops 30 years previously. This had left him with a rash that improved a few days later.

The patient was discharged after a straight forward cataract operation on Maxitrol eye drops four times a day.

He was seen the next day for the first post-operative visit, and up until then had used Maxitrol twice to the affected eye.

At that stage the patient's eye was noted to be slightly more injected than usual but no further concerns were raised. Papillae were noted on the palpebral conjunctiva and treatment was continued.

**Figure 1:**
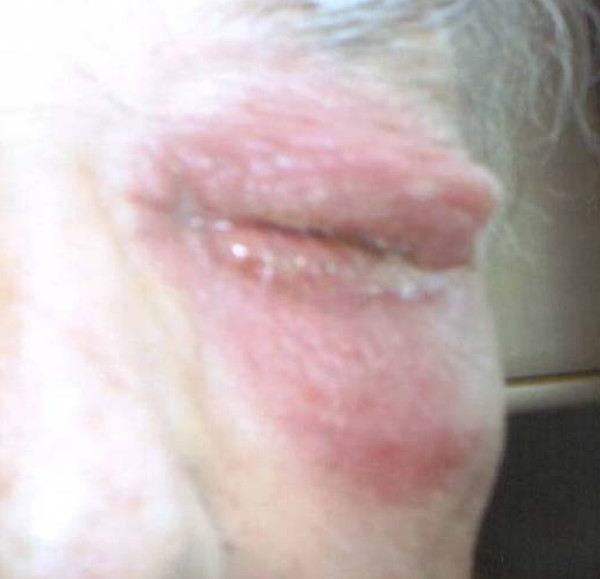
Severe pruritic periorbital rash and swelling around left eye.

**Figure 2:**
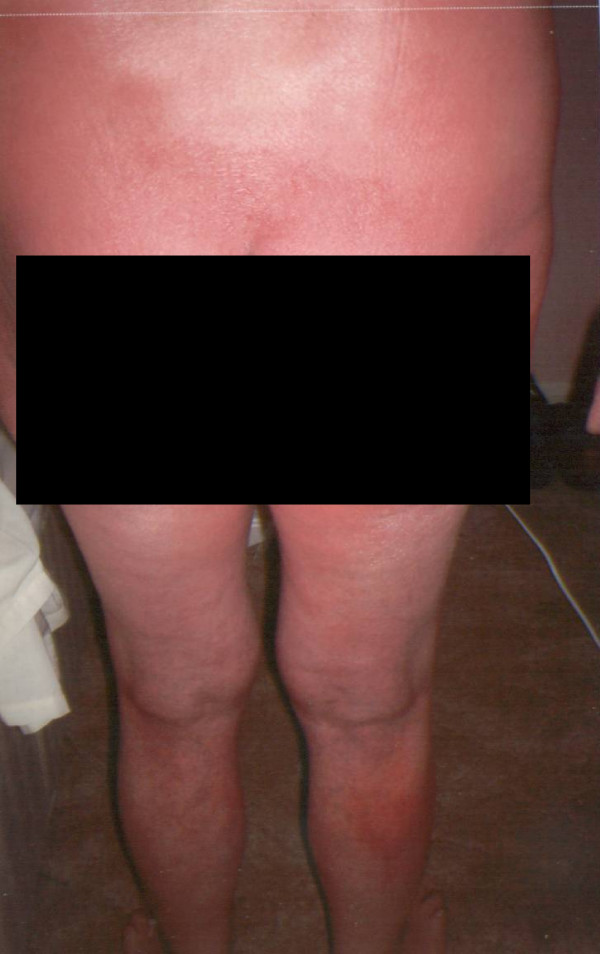
Generalised hypersensitivity reaction involving the trunk and lower limbs.

**Figure 3:**
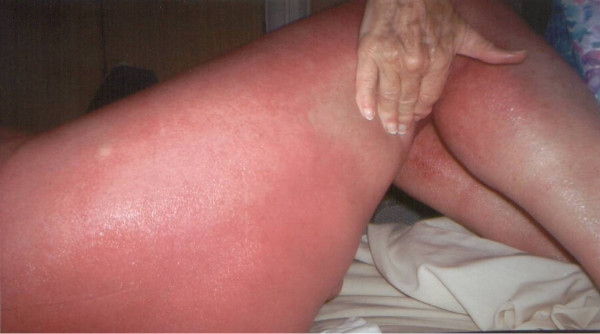
Maculo-papular rash – Involvement of the lower limb.

The patient was then reviewed 2 weeks post-operatively and had continued to use Maxitrol up until that time. He was found to have increased facial erythema, swelling and crusting around the lids. (see Fig. 1). He also complained of a severe and generalised rash affecting his legs, arms, back and buttocks which was painful. (Figs. 2 and 3). No other mucus membranes were involved.

Treatment with systemic Prednisone 20 mg once a day and Betnovate eye ointment relieved his symptoms remarkably, and 3 weeks later all signs of inflammation were gone.

## Conclusion

Systemic hypersensitivity reactions to topical ophthalmic treatment occur rarely. but when they do they can be severe as highlighted by this case.

Although the patient in this report had a substantial improvement in best-corrected visual acuity from counting fingers pre-operatively to 6/9 post-operatively, he endured a long and painful recovery which otherwise would have been quick and straight forward. An alternative post-operative regime could have been Pred Forte and Chloramphenicol eye drops four times a day.

This case report emphasises the importance of a thorough drug and allergy history when patients are seen at pre-assessment or clerked in for surgery. Even when drugs are given topically and locally, the possibility of a severe systemic reaction should always be borne in mind even though these are rare. As physicians it is also important for us to educate our patients about the signs and symptoms of such an allergic reaction so the patient will return to us sooner for treatment.

## Competing interests

The author(s) declare that they have no competing interests.

## Authors' contributions

IAA was the main author and is the corresponding author. EO is the co-author. All authors read and approved the final manuscript.

## Consent

Written informed consent was obtained from the patient for publication of this Case report and accompanying images. A copy of the written consent is available for review by the Editor-in-Chief of this journal.
